# Prevalence of CECR1 mutations in pediatric patients with polyarteritis nodosa, livedo reticularis and/or stroke

**DOI:** 10.1186/1546-0096-13-S1-O87

**Published:** 2015-09-28

**Authors:** R Caorsi, A Grossi, A Insalaco, M Alessio, S Martino, E Cortis, A Morreale, F Caroli, A Martini, I Ceccherini, M Gattorno

**Affiliations:** 1 G. Gaslini Institute, 2nd division of Pediatrics, Genova, Italy; 2 G. Gaslini Institute, Department of Genetics, Genova, Italy; 3 Ospedale Pediatrico Bambino Gesù, Department of Pediatrics, Roma, Italy; 4 Ospedale Federico II, Department of Pediatrics, Napoli, Italy; 5 Ospedale Regina Margherita, Department of Pediatrics, Torino, Italy; 6 Ospedale Santa Maria della Stella, Department of Pediatrics, Orvieto, Italy; 7 Universisty of Genova, Department of Pediatrics, Genova, Italy

## Background

Mutations of CECR1 have been recently reported as causative of an inflammatory condition characterized by polyarteritis nodosa, cerebral stroke and immunodeficiency; the clinical manifestations of the disease are heterogeneous with a wide range of severity.

## Objectives

To analyze the prevalence of CECR1 mutations in pediatric patients with polyarteritis nodosa, livedo reticularis and/or stroke.

## Methods

Pediatric patients of Caucasian Italian origin with the following diseases/manifestations were included in the study: i) histologically confirmed polyartiritis nodosa (PAN) or cutaneous polyartiritis nodosa (cPAN), ii) persistent livedo reticularis with elevation of acute phase reactants, iii) ischemic or hemorrhagic strokes with systemic inflammation. Direct sequencing of CECR1 gene (exons 1-9) was performed with Sanger analysis.

## Results

Up to January 2015, 33 patients from 30 families were included in the study. Homozygous or compound heterozygous *CECR1* mutations with deleterious effects (G47R, G47A, P251L, R312X, E328D, T360A, L249P) were detected in 7 patients. A heterozygous causative mutation (G47V) was observed in 2 affected brothers, their father and the unaffected brother; another patient with clinical manifestations consistent with the disease was found to be heterozygous for the Y453C mutation. In the remaining patients common polymorphisms (L46L, N53N, H335R, Y453Y) were detected.

The mean age of onset of the disease in genetically confirmed patients was 24 months (range 6 months - 5 years); all of them presented fever, elevation of acute phase reactants, livedo reticularis and a skin biopsy suggestive for vasculitis; two of them presented subcutaneous nodules while one of them presented ulcerations at extremities. Hypertension was detected in four patients, while one presented miocarditis. 3 patients presented one or more cerebral stroke during their disease course, while in 3 patients peripheral neuropathy was detected. 4 patients presented intestinal involvement (ranging from recurrent abdominal pain to intestinal perforation) and 2 patients presented growth delay, independent from steroidal treatment. Low immunoglobulin levels were detected in two patients.

The clinical characteristics of the heterozygous patients were similar: fever, livedo reticularis, increased acute phase reactants and hypogammaglobulinemia; cerebral stroke occurred in one of them.

## Conclusions

CECR1 mutations are present in the Italian population and associated with severe cases of ADA2 deficiency. A clinical heterogeneity has been detected in genetically confirmed patients. In a few patients a typical phenotype was associated to incomplete or negative genotype, thus supporting the hypothesis of a genetic heterogeneity of this condition.

